# A Randomized Crossover Pilot Study of Telemedicine Delivered via iPads in Parkinson's Disease

**DOI:** 10.1155/2019/9403295

**Published:** 2019-01-06

**Authors:** Satoko Sekimoto, Genko Oyama, Taku Hatano, Fuyuko Sasaki, Ryota Nakamura, Takayuki Jo, Yasushi Shimo, Nobutaka Hattori

**Affiliations:** ^1^Department of Neurology, Juntendo University School of Medicine, Tokyo 113-8421, Japan; ^2^Research and Therapeutics for Movement Disorders, Juntendo University School of Medicine, Tokyo 113-8421, Japan

## Abstract

**Background:**

We investigated the feasibility and safety of a video-based telemedicine system, delivered via a tablet, in Parkinson's disease (PD).

**Methods:**

In a randomized, crossover, open-label pilot trial, we compared a telemedicine period (regular visits every two months with intermediate video calls via an iPad mini) with a control period (regular visits every two months), both lasting 6 months. We included 10 patients diagnosed with PD according to the British Brain Bank criteria, aged 20–75 years. The primary outcome was the PD questionnaire summary index (PDQ-39 SI). Secondary outcomes included the Hoehn and Yahr Stage and scores on the Unified PD Rating Scale (UPDRS) part I–IV, Beck Depression Inventory (BDI), and visual analog scale for satisfaction.

**Results:**

Both study periods were completed by 10 patients with PD. Friedman's test revealed that there were no significant differences between the two periods in primary and secondary outcomes (*p* > 0.05). With respect to visual analog scale scores for satisfaction, participants indicated high satisfaction with the telemedicine system. The number of extra hospital visits and phone calls did not differ between the periods. There were no adverse events or side effects.

**Conclusions:**

We observed that a telemedicine system delivered via a tablet could successfully be used by patients as a part of their care. Further studies investigating the use of telemedicine to replace in-person visits are warranted. This trial is registered with UMIN000015536.

## 1. Introduction

Aging is a major risk factor for Parkinson's disease (PD), meaning that the number of patients living with PD is steadily increasing as people live longer [1]. In addition, individuals with PD experience not only motor symptoms but also various nonmotor symptoms, such as cognitive dysfunction, psychiatric symptoms, sleep disturbance, urinary problems, sexual dysfunction, and dysautonomia, all of which affect their quality of life (QOL). These symptoms can fluctuate throughout the day and from day-to-day, thus requiring frequent reassessment. At an advanced disease stage, patients may require device-aided therapy, such as a levodopa-carbidopa intestinal gel or deep brain stimulation, due to motor complications such as the “wearing off” phenomenon and dyskinesia.

Due to the fluctuating symptoms and multiple therapeutic choices, it is preferable for patients with PD to consult a neurologist specializing in PD treatment. In fact, reports suggest that, in patients with PD, care under a neurologist might be associated with a better prognosis [2]. However, access to PD specialists may be limited for some individuals as a result of declining mobility with the disease progression, as well as uneven distribution of these specialists between urban and rural areas and the limited number of neurologists specializing in PD [3]. Thus, access to neurologists is considered an important factor in the management of PD symptoms, but it can be limited due to physiological, economical, and geographical reasons.

With the development of information and communication technology (ICT), telemedicine is gradually being put to practical use. Telemedicine can be classified according to the timing of information exchange as either the store-and-forward type or real-time interaction type. Store-and-forward telemedicine can be applied in the field of medical consultation, imaging diagnosis, and pathological diagnosis [4]. Studies of real-time, interactive telemedicine technology using videoconferencing systems have been conducted in the field of neurological diseases [5], and there has also been an expanding use of telemedicine in movement disorders such as PD (Table 1). In 1993, Hubble et al. [6] initially reported that the Unified Parkinson's Disease Rating Scale (UPDRS) motor scores could be assessed remotely with a video conference system. Limitations in the early days of telemedicine included internet speed and the cost of devices, as well as video quality [5, 7]. However, with technological developments, trials of interactive telemedicine have increased. A previous randomized controlled trial reported that telemedicine allowed valid measurement of activities of daily living, the UPDRS, and the Movement Disorder Society sponsored revision of the UPDRS [8, 9]. Moreover, telemedicine has been shown to be able to evaluate the patient's cognition as well as motor performance [10].

In a longitudinal study, Samii et al. [7] investigated the use of videoconferencing in a patient's home, reporting that participants responded positively and that telemedicine saved travel time and costs. Similarly, live videoconferencing can be implemented at nursing homes and adult daycare centers [11, 12], as well as satellite clinics [13]. Although most of these studies have focused on “return patients” using telemedicine in combination with traditional face-to-face services, telemedicine consultation can also be useful for new patients [14, 15]. Moreover, telemedicine has been shown to be useful for rehabilitation and for conducting long-term follow-up, as well as research visits [16–21]. In addition, several other studies have showed the usefulness of store-and-forward types of telemedicine for PD in limited conditions [22, 23].

Thus, previous studies have reported the usefulness of videoconferencing telemedicine in this field, but the evidence for telemedicine using tablet device in PD is not sufficient. Tablet devices may be more suitable for real-time telemedicine for movement disorders, considering both mobility of the patient and quality of the video [10, 13]. This is in contrast to previous video call systems that required a desktop or laptop PC [7, 8, 11, 14–18, 24]. Smartphones can be also useful, but the size of the screen is crucial. Tablet devices can be combined with some applications to measure movements requiring direct touch from patients, such as handwriting [25], and can be combined with wearable devices via Bluetooth to create body area networks [26]. Tablet-based telemedicine contains the possibility of multiple ways of evaluation and usage, but there is no study that verified the feasibility of a real-time interactive telemedicine system using tablets in PD.

In this study, we conducted a pilot study to investigate the feasibility and safety of an interactive, video-based telemedicine system delivered via a tablet device in PD. In this study, we have chosen the iPad mini as the interface for telemedicine, as it is mobile, low-cost, popular, and easy to use for all ages. We hypothesized that QOL of patients with PD would improve with the use of telemedicine utilizing a tablet device, due to reduced travel burden and increased frequency of care.

## 2. Materials and Methods

### 2.1. Study Design

**Figure 1:**
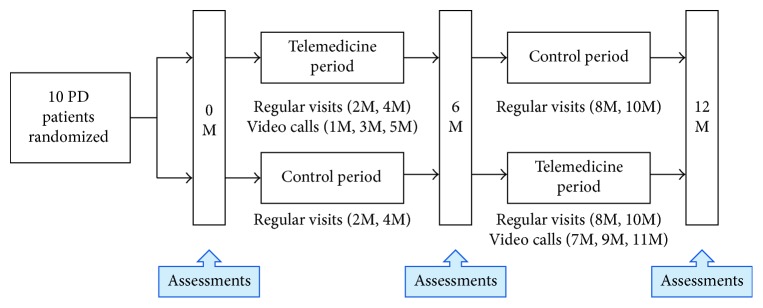
Study design.

We conducted a randomized, crossover, pilot trial comparing a telemedicine period (regular visits every 2 months at 2, and 4 months, or 8 and 10 months with intermediate video calls at 1, 3, and 5 months or 7, 9, and 11 months via an iPad mini, Wi-Fi version, Apple, CA) to a control period (regular visits every 2 months without intermediate video calls) (Figure 1). We use telemedicine visits as a supplement to office visits rather than using them to replace some office visits to ensure the safety of the patients. The institutional review boards at Juntendo University Hospital approved the research protocol and consent forms, and participants provided written consent prior to participation. We included patients diagnosed with PD according to the British Brain Bank criteria [27] (including familial PD), aged 20–75 years, and who have Wi-Fi access at home. Ten patients were recruited from January 2015 to November 2015.

Patients were randomized to receive either the telemedicine period followed by the control period, or the control period followed by the telemedicine period, with both periods lasting 6 months (Figure 1). Simple randomization was carried out using a random number table generated by a computer. The clinicians (GO or TH) were blinded when the participants were randomized into the groups, and then the order of each period was revealed to both the clinicians and patients. During a telemedicine visit, via a video call using the FaceTime app, the clinicians performed a structured interview, medication review, and motor examination, including the modified version of the UPDRS part III, in which the scores of rigidity (item 22) and retropulsion (item 30) were removed [28]. The patient's electrical medical records were available at all telemedicine visits, and all information obtained from the telemedicine visits, as well as regular visits, was recorded in electrical medical records.

To investigate whether telemedicine can improve the QOL of patients with PD, the primary outcome measure was the Parkinson's Disease Questionnaire summary index (PDQ-39 SI), which was measured at baseline, 6 months, and 12 months, in both groups. The secondary outcomes included the Hoehn and Yahr stage and scores on the UPDRS parts I–IV and Beck Depression Inventory (BDI). To compare motor performance between the two periods, the scores of rigidity (item 22) and retropulsion (item 30) were removed from the UPDRS III obtained in the control period. We also evaluated satisfaction with the telemedicine system at 12 months with a visual analog scale (rated 0 to 10).

### 2.2. Statistics

We compared clinical outcomes (PDQ-39 SI, the UPDRS part I–IV, Hoehn and Yahr stage, and BDI) between the telemedicine and control periods with Friedman's test. Data are presented as the median (interquartile range). Background and outcomes of visual analog scale ratings are shown as the mean ± standard deviation. All statistical tests were conducted with the Statistical Package for the Social Sciences (SPSS Version. 23.0; SPSS, Inc., Chicago, IL, US). The significance level was set at *p* < 0.05.

## 3. Results

**Figure 2:**
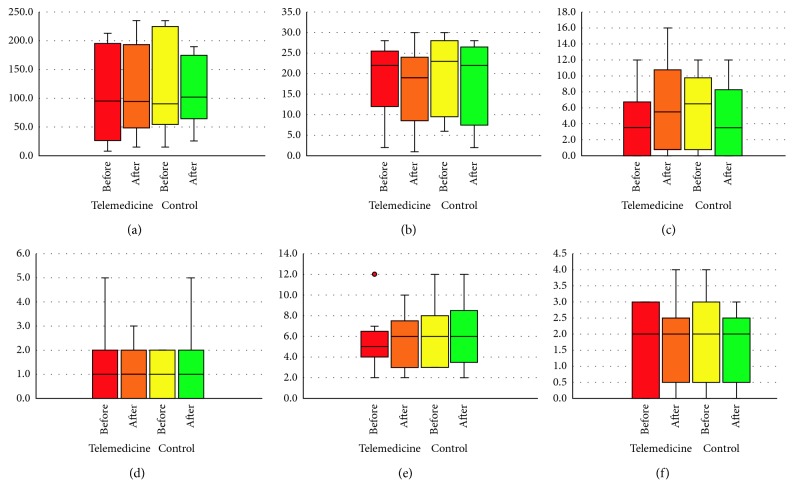
The median (interquartile range) in clinical measures during telemedicine and control periods: (a) PDQ39 SI; (b) UPDRS part III; (c) BDI; (d) UPDRS part I; (e) UPDRS part II; (f) UPDRS part IV.

Both study periods were completed by 10 patients with PD (7 men and 3 women, mean age 53.5 ± 5.5 years). At baseline, patients had a disease duration of 7.3 ± 6.0 years and an average Hoehn and Yahr stage of 2.0 ± 0.5. There were no significant changes in PDQ-39 SI during the telemedicine or control period (*p*=0.87) (Table 2, Figure 2).

Regarding secondary outcomes, we observed no significant change in the UPDRS part III scores in the “ON” state during the telemedicine period or control period (*p*=0.08). There were no significant changes in the UPDRS part I, II, and IV scores (*p*=0.93, 0.89, and  0.93, respectively), modified Hoehn and Yahr stage (*p*=0.26), or BDI scores (*p*=0.64) during the telemedicine period or control period (Table 2, Figure 2). One participant had two extra hospital visits in the telemedicine period and two extra hospital visits in the control period. There were no extra phone calls in either period.

**Figure 3:**
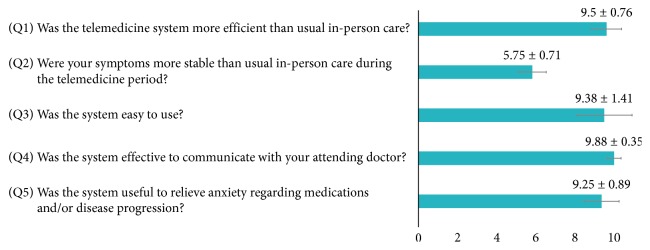
Bar chart of ratings on the visual analog questionnaire measuring patient satisfaction with the telemedicine system.

Participants rated their satisfaction with the telemedicine system with a visual analog scale (Figure 3). Most patients agreed that the tablet-based telemedicine system was easy to use, useful to relieve anxiety regarding medications and/or disease progression, an effective way to communicate with doctors, and more efficient than the usual, in-person care. However, they did not agree that it helped to stabilize symptoms during the study period.

## 4. Discussion

Our results indicate that the outcomes of telemedicine were comparable to those of regular, face-to-face visits and suggest that a tablet device, such as an iPad, is a feasible method to administer telemedicine in real-time situations. Furthermore, ratings on a visual analog scale indicated that the telemedicine system was well-tolerated by patients.

Although it was expected that tablet-based telemedicine could reduce the number of extra hospital visits and phone calls, there was no difference in the number of extra hospital visits and phone calls during each period in our study. This may be because patients who were enrolled in this study had relatively mild symptoms. To confirm this hypothesis, studies including patients in more advanced stage of the disease are required.

The biggest limitation of tablet-based telemedicine is the restriction on evaluations that require in-person contact, similar to previous computer-based videoconferencing systems. For example, measures such as rigidity and postural stability in the UPDRS cannot be evaluated without physical interaction. However, it has been shown that a modified UPDRS, excluding rigidity and postural stability, is not inferior to face-to-face evaluation using the conventional UPDRS [28]. In addition, combining telemedicine with wearable devices containing accelerometers, gyroscopes, magnetometers, and global positioning systems may overcome this limitation.

The second limitation of tablet-based telemedicine is the quality and resolution of the video. For example, subtle and quick involuntary movements, characterizing mild dyskinesia and small-amplitude tremor, may be difficult to detect. However, these limitations can be minimized with future technological advancements. Furthermore, this could be improved by transferring not only two-dimensional video imaging, but also three-dimensional motion images and data.

The third limitation of tablet-based telemedicine is a concern regarding the doctor-patient relationship [29]. Indeed, it has been reported that some participants in previous telemedicine studies preferred in-person visits. However, in our study, patients reported high degrees of satisfaction, which is consistent with several previous studies [7, 12, 19, 30], as well as a preference for remote visits [12, 24]. High satisfaction has been reported for the use of telemedicine among new patients, even when used alone [15]. Therefore, at the very least, telemedicine visits could be used in practice to complement conventional care. Furthermore, telemedicine may have additional benefits, beyond the traditional doctor-patient relationship, such as allowing direct communication among experts, caregivers, and local primary-care providers. It may also allow experts to develop closer relationships with patients, provide education, and share information more easily [7].

Finally, there are some limitations specifically related to this study, including the open-label design, small sample size, and short time span. A total duration of 6 months may be too short a time frame, and follow-up visits every 2 months in each period too frequent, to detect changes in quality of life and motor symptoms. In addition, the lack of a washout period in this study could have caused carryover effects. Finally, recruitment of relatively young patients, which may cause selection bias, may not be suitable for detecting changes in outcome with a short study period.

## 5. Conclusions

This pilot study showed that a telemedicine system delivered via a tablet was successfully and safely used by patients with PD. Further studies investigating the use of telemedicine to replace in-person visits are warranted. Further studies are also needed to support the validity of telemedicine as a method of care for patients, including those in the advanced stages of PD. Such systems may be particularly useful for patients who are living at long distances from, and are unable to travel to, a specialist center, reducing both the cost and travel burden for such patients. For practical realization of telemedicine, the limitations regarding reimbursement, as well as those related to technical reliability and confidentiality, should be addressed.

## Figures and Tables

**Table 1:** The literatures of telemedicine using the real-time videoconferencing system.

Authors	Year	*N*	Study design	System	Result
Hubble et al. [6]	1993	9	Nonrandomized, controlled (one time, 90 min time interval)	Motor assessment by the video conference system	The UPDRS score by the system was comparable with face-to-face assessment
Samii et al. [7]	2006	34	Longitudinal observational (follow-up care, 3 years)	Videoconferencing system at home	Participants rated the questionnaire positively, telemedicine saved travel time and cost
Hoffmann et al. [8]	2008	12	Randomized, controlled (one-time, simultaneous evaluation)	Evaluation of motor and ADL via computer-based video conference system	The system was valid to measure ADL and the UPDRS. Intra- and interrater reliabilities were high
Tindall et al. [17]	2008	24	Case series (4 days/week, 4 weeks)	Delivery of LSVT via videophone	Vocal loudness improved; satisfaction with the technology combined to make videophone-delivered therapy
Biglan et al. [11]	2009	1	Case report (6 visits/8 months)	Live videoconferencing at the nursing home	Improvement in motor and cognitive symptoms (fewer dyskinesia, longer on time, improved MMSE); patient satisfied with the care
Fincher et al. [14]	2009	36	Randomized, controlled (one-time medication consultation)	Medication consultation via desktop videophone (21) vs telephone (15)	Videophones were significantly useful than telephones; satisfied more with videophones
Howell et al. [16]	2009	3	Case series (3 times/week, 4 weeks)	Internet delivery of LSVT	The system was comparable
Constantinescu et al. [18]	2010	1	Case report (4 days/week, 4 weeks)	Delivery of LSVT via PC-based videoconferencing system	Improvements in voice; satisfied with overall treatment
Dorsey et al. [12]	2010	10	Randomized, controlled (4 visit/6 months)	Telemedicine visit to the nursing home and adult day care populations via notebook computer-based videoconferencing telemedicine care (6) vs usual care (4)	Significant improvements in QOL and the UPDRS motor scale
Constantinescu et al. [19]	2011	34	Randomized, controlled (4 days/week, 4 weeks)	Online delivery of LSVT via PC-based videoconferencing system	Noninferiority of the online LSVT modality was confirmed; high participant satisfaction was reported overall
Dorsey et al. [24]	2013	20	Randomized, controlled, two centers (7 months)	Web-based videoconferencing (“virtual house call”) telemedicine (9) vs in-person care (11)	The change in quality of life did not differ
Venkataraman et al. [15]	2014	35	Case series (one time)	Specialist consultation for new patients via videoconferencing system	Patients satisfaction exceeded 90%
Qiang and Marras [30]	2015	34	Retrospective controlled study	Satisfaction questionnaire was compared between the previous user of video-based telemedicine use (34) vs nonuser (103)	Telemedicine reduced the cost; patients preferred combination of telemedicine and in-person visit
Dorsey et al. [20]	2015	166	Observational	Virtual research visit using web cameras and videoconferencing software	Overall satisfaction with visit was 79% (neurologists) and 93% (participants)
Stillerova et al. [10]	2016	11	Nonrandomized, self-controlled (one time)	MoCA test via video conference system (Skype; PC9, smartphone/tablet2)	Result is not different; system was viable
Stillerova et al. [9]	2016	11	Nonrandomized, controlled (one time)	Face-to-face vs videoconferencing software (Skype or Google Hangouts) using computers and webcams for evaluation of the MDS-UPDRS	Internet-based videoconferencing may be useful
Wilkinson et al. [13]	2016	86	Dual-arm, randomized, controlled (12 month)	Video telehealth visit home arm: 18 actives (tablet-PC), 18 controls; satellite clinic arm: 26 actives (Carts and web-come), 24 controls	High patient satisfaction, reduced travel burden, equal clinical outcomes
Barbour et al. [21]	2016	16	Long-term observational (over 3 years)	Videoconferencing system	Participants, families, subspecialists, and the nursing staff expressed uniformly high satisfaction

MMSE: Mini-Mental State Examination; LSVT: Lee Silverman Voice Treatment; MoCa: Montreal Cognitive Assessment.

**Table 2:** Changes in clinical measures during telemedicine and control periods (median, interquartile range, and *p* value).

	Telemedicine period		Control period		*p* value
	Baseline	6 months	Baseline	6 months	
PDQ39 SI	95.45 (26.45–195.425)	94.45 (48.45–193.15)	90.4 (54.375–224.725)	102.1 (64.35–174.675)	0.87
UPDRS part III	22.0 (12.0–25.5)	19.0 (8.5–24.0)	23.0 (9.5–28.0)	22.0 (7.5–26.5)	0.08
BDI	3.5 (0–6.75)	5.5 (0.75–10.75)	6.5 (0.75–9.75)	3.5 (0–8.25)	0.64
UPDRS part I	1.0 (0–2.0)	1.0 (0–2.0)	1.0 (0–2.0)	1.0 (0–2.0)	0.93
UPDRS part II	5.0 (4.0–6.5)	6.0 (3.0–7.5)	6.0 (3.0–8.0)	6.0 (3.5–8.5)	0.89
UPDRS part IV	2.0 (0–3.0)	2.0 (0.5–2.5)	2.0 (0.5–3.0)	2.0 (0.5–2.5)	0.93
Modified Hoehn and Yahr stage	2.0 (2.0–2.0)	2.0 (2.0–2.0)	2.0 (2.0–2.0)	2.0 (1.5–2.0)	0.26

## Data Availability

The datasets used and/or analyzed during the current study are available from the corresponding author on reasonable request.
